# Supplementary material for: A machine-readable specification for genomics assays
Source: Bioinformatics. 2024 Apr 5;40(4):btae168. doi: 10.1093/bioinformatics/btae168 (PMC11009023; doi:10.1093/bioinformatics/btae168)
Supplement: btae168_Supplementary_Data [file btae168_supplementary_data.docx]

**Supplementary Information**:

A machine-readable specification for genomics assays

A. Sina Booeshaghi^1*^, Xi Chen^2^, and Lior Pachter^1,3*^

1. Department of Bioengineering, University of California, Berkeley, California
2. School of Life Sciences, Southern University of Science and Technology, Shenzhen, China
3. Department of Computing and Mathematical Sciences, California Institute of Technology, Pasadena, California

*Address correspondence to [sinab@berkeley.edu](mailto:sinab@berkeley.edu) & [lpachter@caltech.edu](mailto:lpachter@caltech.edu)


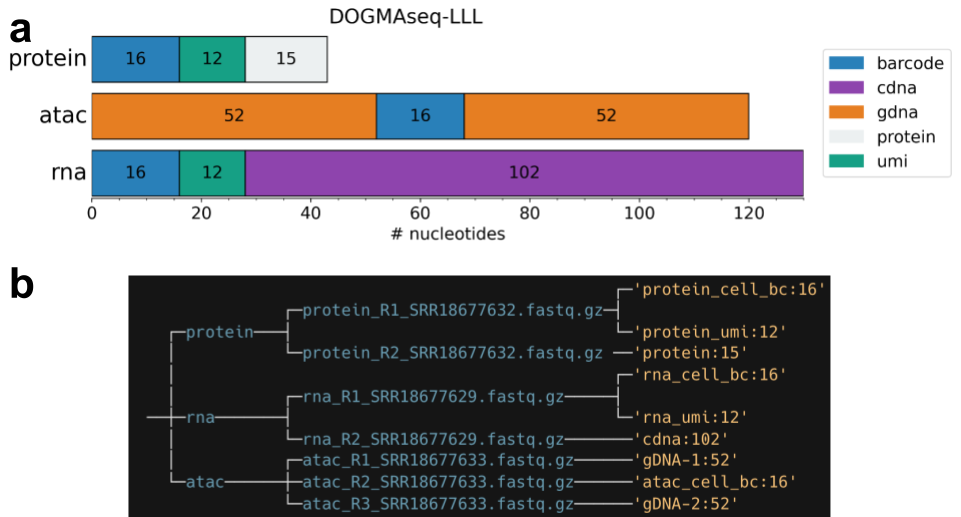


**Supplementary Figure 1:** *seqspec* read structure of the DOGMAseq-LLL [(Xu et al. 2022)](https://paperpile.com/c/e0GH3V/P61S) assay annotated by (a) atomic regions and their lengths. The (b) ordered-tree representation of the reads.

**References**

[Xu, Zhongli, Elisa Heidrich-O’Hare, Wei Chen, and Richard H. Duerr. 2022. “Comprehensive Benchmarking of CITE-Seq versus DOGMA-Seq Single Cell Multimodal Omics.” *Genome Biology* 23 (1): 1–17.](http://paperpile.com/b/e0GH3V/P61S)
